# The downside of metabolic diversity: Postingestive rearrangements by specialized insects

**DOI:** 10.1073/pnas.2122808119

**Published:** 2022-06-06

**Authors:** Sven Heiling, Jiancai Li, Rayko Halitschke, Christian Paetz, Ian T. Baldwin

**Affiliations:** ^a^Department of Molecular Ecology, Max Planck Institute for Chemical Ecology, 07745 Jena, Germany;; ^b^CAS Key Laboratory of Insect Developmental and Evolutionary Biology, Center for Excellence in Molecular Plant Sciences, Shanghai Institute of Plant Physiology and Ecology, Chinese Academy of Sciences, Shanghai, 200032 China;; ^c^Department of Biosynthesis/NMR, Max Planck Institute for Chemical Ecology, 07745 Jena, Germany

**Keywords:** plant-specialized metabolites, frass metabolites, synergy effects, chlorogenic acid, diterpenoids

## Abstract

Deploying toxins in complex mixtures is thought to be advantageous and is observed during antagonistic interactions in nature. Toxin mixtures are widely utilized in medicine and pest control, as they are thought to slow the evolution of detoxification counterresponses in the targeted organisms. Here we show that caterpillars rearrange key constituents of two distinct plant defense pathways to postingestively disable the defensive properties of both pathways. Specifically, phenolic esters of quinic acid, chlorogenic acids (CAs), potent herbivore and ultraviolet (UV) defenses, are reesterified to decorate particular sugars of 17-hydroxygeranyllinalool diterpene glycosides (HGL-DTGs) and prevent their respective anti–herbivore defense functions. This was discovered through the employment of comparative metabolomics of the leaves of *Nicotiana attenuata* and the frass of this native tobacco’s specialist herbivore, *Manduca sexta* larvae. Feeding caterpillars on leaves of transgenic plants abrogated in each of the two pathways, separately and together, revealed that one of the fully characterized frass conjugates, caffeoylated HGL-DTG, originated from ingested CA and HGL-DTGs and that both had negative effects on the defensive function of the other compound class, as revealed by rates of larval mass gain. This negative defensive synergy was further explored in 183 *N. attenuata* natural accessions, which revealed a strong negative covariance between the two defense pathways. Further mapping analyses in a biparental recombinant inbred line (RIL) population imputed quantitative trait loci (QTLs) for the two pathways at distinct genomic locations. The postingestive repurposing of defense metabolism constituents reveals a downside of deploying toxins in mixtures, a downside which plants in nature have evolved to counter.

## Significance

Higher plants produce specialized metabolites to cope with biotic and abiotic challenges faced in natural environments. The diversity and complexity of specialized metabolism is often beneficial, providing functional synergisms and evolutionary stability when metabolic solutions to ecological problems are deployed as mixtures. These benefits of diversity have recently been realized in the deployment of antibiotics, insecticides, fungicides, and herbicides. However, the functional downside of metabolite diversity is rarely studied, in part because the mechanisms of post-ingestive metabolite interactions are largely unknown. Here we showed that larvae of the tobacco hornworm, *Manduca sexta*, rearrange key constituents of two distinct defense pathways in its wild tobacco host plant, *Nicotiana attenuata*, to thwart the defensive properties of both pathways.

Being at the foundation of most food webs on the planet, autotrophic plants require sophisticated defenses to survive in a world full of heterotrophs. Chemicals allow plants to cope not only with attack from heterotrophic organisms (1) but also with abiotic challenges (drought, extreme temperature, and irradiation) (2) and even to communicate with neighbors (3). Plants have evolved diverse specialized metabolomes to solve these ecological challenges. Several influential hypotheses invoke functional interpretations for this metabolite diversity, such as increasing the likelihood of producing a bioactive compound against a natural enemy (4) and providing the functional redundancy that facilitates the evolution of compounds that target specific natural enemies (5). In short, producing multiple specialized metabolites often increase a plant’s Darwinian fitness in environments with diverse natural enemies (5, 6). The responsible mechanisms for the adaptive value of metabolic diversity are also diverse, including defensive synergisms (7) and the evolutionary stability that mixtures provide in resistance management of insecticides, herbicides, and antibiotics in the face of coevolving antagonists. However, the possible functional downsides of diversity have received less attention.

Specialist insect herbivores that have evolved the ability to feed on only a few closely related host plant taxa provide ideal natural systems with which to study functional interactions among diverse plant-specialized metabolites. These specialist herbivores have evolved multiple adaptive mechanisms that allow them to degrade and detoxify ingested defenses, avoid contact and ingestion, evolve target-site insensitivity, rapidly excrete ingested defenses, and even sequester plant defenses to co-opt them for their own defense (8, 9). For example, although glucosinolates, the main chemical defense of Brassicaceae, are hydrolyzed to release toxic isothiocyanates in response to attack, many insects readily feed and perform well on glucosinolate-accumulating plants by thwarting glucosinolate breakdown or deactivating toxic products (10, 11). Whether these diverse plant defenses interact after ingestion remains poorly studied.

The wild tobacco, *Nicotiana attenuata*, an ecological model plant with a rich portfolio of specialized metabolites, has long interacted with the voracious larvae of the lepidopteran *Manduca sexta*, a specialist that feeds largely on Solanaceae plants. *N. attenuata* plants accumulate large quantities of defensive pyridine alkaloids, proteinase inhibitors, 17-hydroxygeranyllinalool diterpene glycosides (HGL-DTGs), phenolamides, and phenolics (12–16) and hence constitute an excellent system in which to explore functional interactions among diverse specialized metabolites. Some of these interactions can result in defensive synergies, as has been shown with the neurotoxin nicotine and the antidigestive defense of proteinase inhibitors, where nicotine prevents herbivores from consuming more plant material to compensate for the effects of the proteinase inhibitors (17).

Here, a metabolomic comparison of *N. attenuata* leaves and *M. sexta* larval frass revealed a class of compounds derived from quinic acid conjugates and HGL-DTGs, plant defenses which are synthesized through two distinct pathways. Performance assays revealed that this specialist herbivore produced the compounds in the process of counteracting the defensive function of the two distinct defense pathways. A strong negative genetic covariance in the regulation of these two plant defense pathways was uncovered in natural accessions and a recombinant inbred population, suggesting that this herbivore’s detoxification strategy has influenced selection for metabolic diversity in its hostplant.

## Results and Discussion

### Compounds Appear in the Frass of Insect Herbivores.

The coyote tobacco, *N. attenuata*, is native to the Great Basin Desert in the southwestern United States and is an ecological model used to study the function of different sectors of specialized metabolism. HGL-DTGs are a class of abundant defensive specialized metabolites in *Nicotiana* plants (13), and a recent study revealed that metabolomic analyses of herbivore frass provided a robust means of understanding the defensive mechanisms and cellular targets of ingested HGL-DTGs (18). To better understand the metabolism of HGL-DTGs in herbivore guts, we employed a tandem mass spectrometry (MS/MS) analytical dereplication workflow (19) to compare the metabolomes of *N. attenuata* leaves and the frass of a specialist herbivore, *M. sexta* larvae, feeding on the leaves of this native tobacco.

**Fig. 1.**
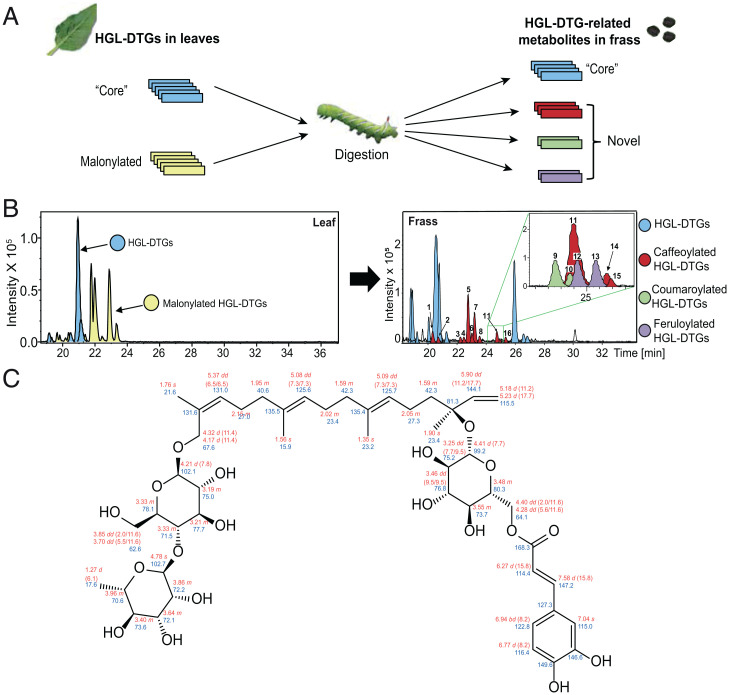
HC-HGL-DTGs appear in the frass of *M. sexta* larvae that fed on *N. attenuata* plants. (*A*) The scheme of the comparative metabolomics analysis of the leaves of *N. attenuata* and frass of *M. sexta* larvae fed on *N. attenuata* leaves. (*B*) Comparative chromatographic analysis between leaves and *M. sexta* frass confirmed the loss of malonyl moieties of HGL-DTGs and revealed a class of HC-HGL-DTGs in *M. sexta* frass that are not detected in the leaves ingested by the larvae. All the detected HC-HGL-DTGs were numbered following their retention times, and their corresponding annotated mass spectra are shown in *SI Appendix*, Fig. S1. (*C*) The NMR-verified chemical structure of the peak 5 HC-HGL-DTG, which is a caffeoylated HGL-DTG. Red: ^1^H chemical shifts δ (ppm, multiplet, ^3^J_HH_ in hertz). Blue: ^13^C chemical shifts (δ ppm).

Consistent with previous research, this comparative analysis revealed that the malonyl moieties of HGL-DTGs were lost in the alkaline condition of the larval midgut (20) and all malonylated HGL-DTGs were converted into the core glycosides (Fig. 1 *A* and *B*). In addition to the previously identified 3-O-α-rhamnopyranosyl-(1→4)-β-glucopyranosyl-17-hydroxygeranyllinalool (RGHGL), a deglycosylation and detoxification product of HGL-DTGs (20), we detected a group of unknown metabolites with the diagnostic fragment corresponding to the aglycone of HGL-DTGs (*SI Appendix*, Fig. S1*A*). Our rapid dereplication and identification workflow detected three known fragments diagnostic of hydroxycinnamoyl (HC) derivatives: *mass-to-charge* ratio (*m/z)* 163.0389 ([M+H]^+^) corresponding to caffeoyl, *m/z* 147.0440 ([M+H]^+^) corresponding to coumaroyl, and *m/z* 177.0546 ([M+H]^+^) corresponding to feruloyl, as previously verified (21). A detailed mass spectrometry (MS) analysis and putative structure descriptions suggested that the HC moieties were linked to the sugar moieties of the HGL-DTGs to form hydroxycinnamoylated HGL-DTGs (HC-HGL-DTGs) (*SI Appendix*, Fig. S1*B*). To test this inference, we purified one of the most abundant peaks (peak 5 in Fig. 1*B*) from the frass of *M. sexta* larvae that fed on *N. attenuata* plants and conducted NMR analysis, which confirmed the structure as a caffeoylated HGL-DTG (Fig. 1*C*).

### Moieties of the Compounds Are from Two Distinct Plant Defense Pathways.

The HGL-DTG part of HC-HGL-DTGs likely originates from plant-produced HGL-DTGs, a diverse metabolic sector of *N. attenuata*. To test this inference, we fed *M. sexta* larvae several lines of transformed plants silenced in different HGL-DTG biosynthetic genes by RNA inference, including geranylgeranyl pyrophosphate synthase (irGGPPS) (22) and uridine diphosphate glycosyltransferases (irUGT74P5, irUGT74P3&5, and irUGT91T1) (23), and compared their frass with that from larvae fed wild-type (WT) *N. attenuata* plants. In all cases, the abundances of HC-HGL-DTGs in the frass were dramatically decreased when larvae fed on HGL-DTG–depleted plants (*SI Appendix*, Fig. S2*A*), demonstrating the plant-based origin of HGL-DTG moieties and leading to the question of the origin of the HC part of the HC-HGL-DTGs in frass.

**Fig. 2.**
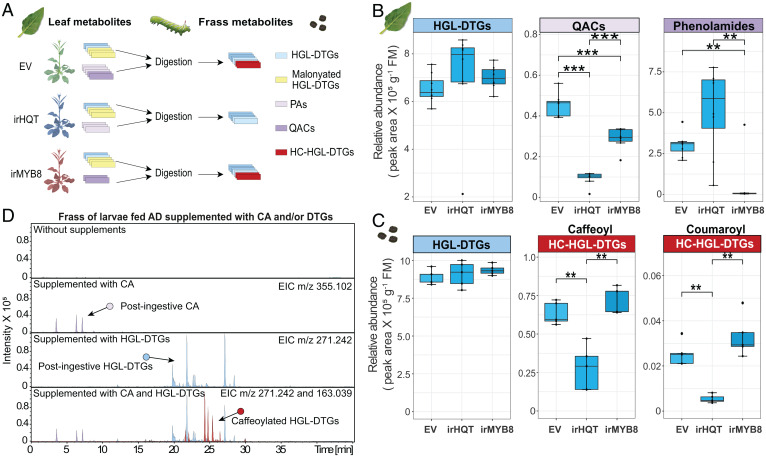
The caffeoyl moieties of caffeoylated HGL-DTGs arise from ingested CAs. (*A*) Specialized metabolomes of leaves of three different transgenic wild tobacco plants were compared with the frass metabolomes of caterpillars fed these leaves; different colors reflect different classes of metabolites; PA, phenolamides. (*B*) The concentrations ( *n* = 6, in fresh mass (FM)) of HGL-DTGs (blue), quinic acid conjugates (QACs; pink), and phenolamides (purple) in homozygous transgenic plants that were silenced in CA accumulations [irHQT (15)] or in the expression of the phenolamide master regulator [irMYB8 (14)], with plants transformed with an EV as controls. (*C*) The concentrations (*n* = 6) of HGL-DTGs and two major forms of HC-HGL-DTGs (red), caffeoylated and coumaroylated, in the frass of *M. sexta* larvae fed different transgenic plants. (*D*) Supplementing AD with CA and HGL-DTGs results in the accumulation of caffeoylated HGL-DTGs in the frass of *M. sexta* larvae feeding on these diets. Asterisks indicate significant differences compared with data from EV plants (**P < 0.01; ***P < 0.001; Student *t* test with Bonferroni correction). (*B* and *C*) The upper whisker extends from the hinge to the largest value no further than 1.5 * interquartile range (IQR) from the hinge and the lower whisker extends from the hinge to the smallest value at most 1.5 * IQR of the hinge.

*N. attenuata* plants produce two major classes of compounds that contain HC moieties: caffeoylquinic acids and phenolamides. To parse the potential origins of the HC moieties, we fed *M. sexta* larvae leaves of transgenic plants silenced in the expression of HC–coenzyme A (CoA) quinate transferase (irHQT), which controls the last step of the conjugation between HC moieties and quinic acid (15), or the phenolamide master regulatory transcription factor *NaMYB8* (irMYB8) (14) and quantified metabolites in larval frass (Fig. 2*A*). While both lines of transgenic plants have WT levels of HGL-DTGs in their leaves, the contents of quinic acid conjugates and phenolamides were specifically regulated in irHQT and irMYB8 leaves, respectively (Fig. 2*B* and *SI Appendix*, Fig. S2 *B* and *C*). HC-HGL-DTG levels were dramatically decreased only in the frass of larvae that were fed irHQT plants, not of those fed irMYB8 plants, although the levels of core HGL-DTGs in the frass were not significantly affected (Fig. 2*C* and *SI Appendix*, Fig. S2 *B* and *C*). From these results, we inferred that the origin of the HC moieties is from ingested HC quinate conjugates.

To further evaluate the origins of HC-HGL-DTGs, we fed *M. sexta* larvae artificial diet (AD) supplemented with a caffeoyl quinate ester, chlorogenic acid (CA); HGL-DTGs; or both. The HC-HGL-DTGs were only detected in frass of larvae fed AD supplemented with both CA and HGL-DTGs (Fig. 2*D*). Notably, CA can isomerize to cryptochlorogenic acid and neochlorogenic acid when ingested by *M. sexta* larvae, likely due to the alkaline environment of the larval midgut (*SI Appendix*, Fig. S3*A*). Alkaline buffer alone can isomerize CA but does not lead to the production of HC-HGL-DTGs (*SI Appendix*, Fig. S3*B*). Additionally, HC-HGL-DTGs accumulate mainly in the gut content, followed by hindgut and midgut body tissues (*SI Appendix*, Fig. S3 *C–E*). From these results, we inferred that HC-HGL-DTGs are postingestively produced from ingested CA and HGL-DTGs, and their conjugation is likely an active process controlled by the larvae, considering that *M. sexta* lacks a resident gut microbiome (24).

### Postingestive Conjugation Thwarts Defense Functions of Two Distinct Pathways.

**Fig. 3.**
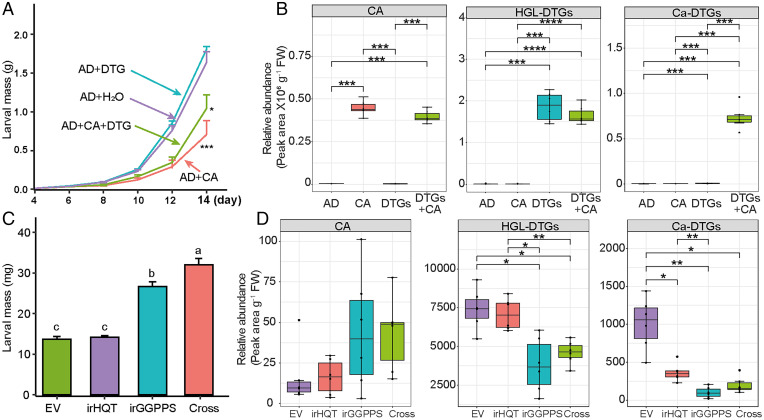
*M. sexta* larvae use CA and HGL-DTGs to detoxify each other. (*A*) Mass (mean + standard error (SE), *n* = 13–15) of *M. sexta* larvae fed AD supplemented with 1.2 mg g^−1^ CA, 2 mg g^−1^ HGL-DTGs (also abbreviated DTG or DTGs in the figure image), or both CA and HGL-DTGs, compared with AD supplied with only water (H_2_O) as control (Con). (*B*) Relative abundances (*n* = 5) of CA, HGL-DTGs, and caffeoylated DTGs (Ca-DTGs) in the frass of *M. sexta* larvae that fed on different AD. (*C*) Mass (mean + SE, *n* = 36–40) of *M. sexta* larvae fed hemizygous transgenic irHQT, irGGPPS plants, or an irHQT x irGGPPS Cross for 4 d. (*D*) The relative abundances (*n* = 5) of CA, HGL-DTGs, and Ca-DTGs in the frass of *M. sexta* larvae that fed on different transgenic plants. Asterisks indicate significant differences among different groups (*P < 0.05; **P < 0.01; ***P < 0.001; ****P < 0.0001; Student *t* test with Bonferroni correction). (*B* and *D*) The upper whisker extends from the hinge to the largest value no further than 1.5 * interquartile range (IQR) from the hinge and the lower whisker extends from the hinge to the smallest value at most 1.5 * IQR of the hinge.

Semiquantitative comparisons of HC-HGL-DTGs and HGL-DTGs in the frass of *M. sexta* larvae that fed on *N. attenuata* plants revealed that about 10% of ingested HGL-DTGs are conjugated to produce HC-HGL-DTGs (Fig. 2*C*). Considering the large amounts of HGL-DTGs in *N. attenuata* aboveground tissues [∼6 mg g^−1^ fresh mass (20)], HC-HGL-DTGs are quantitatively abundant in frass. To investigate the defensive function of this conjugation, we performed a larval performance bioassay with AD supplemented with CA, HGL-DTG, or both compounds. CA supplementation significantly decreased larval growth; this effect was marginally, but not significantly, attenuated when both HGL-DTGs and CA were added into AD (Fig. 3*A*). Consistent with the qualitative analysis, the caffeoylated HGL-DTGs only accumulated in the frass of *M. sexta* larvae that fed on AD supplemented with both CA and HGL-DTGs (Fig. 3*B*). Frass CA levels were slightly decreased when larvae were fed AD supplemented with both CA and HGL-DTGs, compared with that of larvae fed AD supplemented only with CA (Fig. 3*B*). From these data, we inferred that ingested HGL-DTGs can detoxify ingested CA. Consistent with previous research that the defensive function of HGL-DTGs requires their metabolism into hydroxylated derivatives, a process not occurring when larvae feed on AD (18), HGL-DTG supplementation of AD did not affect *M. sexta* larval growth (Fig. 3*A*). Although AD have long been used to evaluate defensive functions of purified metabolites or plant extracts, their drawbacks are well described. Insect AD not only contain large quantities of antibiotics and antioxidants to enhance their shelf life, but they are also commonly based on unnatural components (wheat germ and milk protein) that provide larvae with unrealistically high protein/carbohydrate ratios, which are known to influence a larva’s ability to detoxify defense metabolites (25). Thus, performance bioassays with plants genetically modified in their metabolite accumulations provide a superior means of evaluating the defensive function of specialized metabolites.

To parse functional consequences of the interaction between HGL-DTGs and CA in the natural dietary context of a plant, we conducted larval performance assays on irGGPPS plants which are silenced in HGL-DTG production, irHQT plants which are silenced in CA production, the hemizygous cross of both defense pathway-silenced lines (Cross), and empty vector (EV) plants as control so as to provide plant diets in which the HGL-DTGs and CA were manipulated independently or simultaneously (*SI Appendix*, Fig. S4). Consistent with previous research, HGL-DTGs proved to be one of the most robust defensive compounds in *N. attenuata* (26): *M. sexta* larvae fed irGGPPS plants grew much better than larvae fed EV plants (Fig. 3*C*). There are three copies of *NaGGPPS* in the *N. attenuata* genome, with the copy that dominates HGL-DTG synthesis specifically silenced in irGGPPS plants (22). To further test the inference about the defensive function of HGL-DTGs, we performed *M. sexta* larval bioassays on virus-induced gene-silenced (VIGS) plants, in which the expression of the cytochrome P450 gene (*NaCYP736A*) that controls a key branch point in HGL-DTG biosynthesis was silenced (18). Similar to their performance on irGGPPS plants, larvae grew much better on *NaCYP736A*-silenced plants than on EV plants. The HC-HGL-DTG contents of the frass of *M. sexta* larvae that fed on different transgenic plants followed the same pattern as another detoxification product, RGHGL (*SI Appendix*, Fig. S5). These results demonstrate the defensive function of HGL-DTGs in planta.

In contrast, the defensive function of CA, while apparent in the experiments conducted with supplemented AD (Fig. 3*A*), was not apparent when comparing larvae fed EV and irHQT plants. This result is consistent with the results of a series of earlier experiments with CA biosynthesis-modified transgenic *Nicotiana tabacum* plants, which revealed no negative effects of CA on larval growth (27–29). However, *M. sexta* larvae grew significantly better on the Cross plants than on the irGGPPS plants, indicating a context-dependent defensive function of CA in planta. The different performances of larvae fed EV vs. irHQT plants and larvae fed irGGPPS vs. Cross plants suggest an interaction between CA and HGL-DTGs after ingestion. In contrast with the HGL-DTG–producing *Nicotiana* plants, CA’s defensive function has been demonstrated in chrysanthemum plants which do not produce HGL-DTGs (30). Taken together, these in planta bioassays reveal that the defensive function of CA is thwarted when HGL-DTGs are present, likely by their use in the detoxification of HGL-DTGs to form HC-HGL-DTGs. The trend of increased CA in the frass of larvae feeding on irGGPPS and Cross plants is consistent with this inference (Fig. 3*D*). In addition, the hydroxylated products of HC-HGL-DTGs, which are the defensive form of HGL-DTGs (18), were not detected in the frass (*SI Appendix*, Fig. S3*F*). Hence, the apparent lack of CA defensive function is the integrated outcome of CA’s defensive and detoxification functions. Previously, the quinic acid moieties of CA were reported as conjugated to saligenin, a defensive phenolic glucoside in salicaceous plants, in the frass of another lepidopteran specialist herbivore (31), suggesting that several lepidopteran herbivores can use CA as a versatile detoxification agent. In short, the specialist herbivore *M. sexta* uses two distinct defensive compounds to detoxify each other.

### Natural Populations Revealed the Signatures of Counterresponses.

**Fig. 4.**
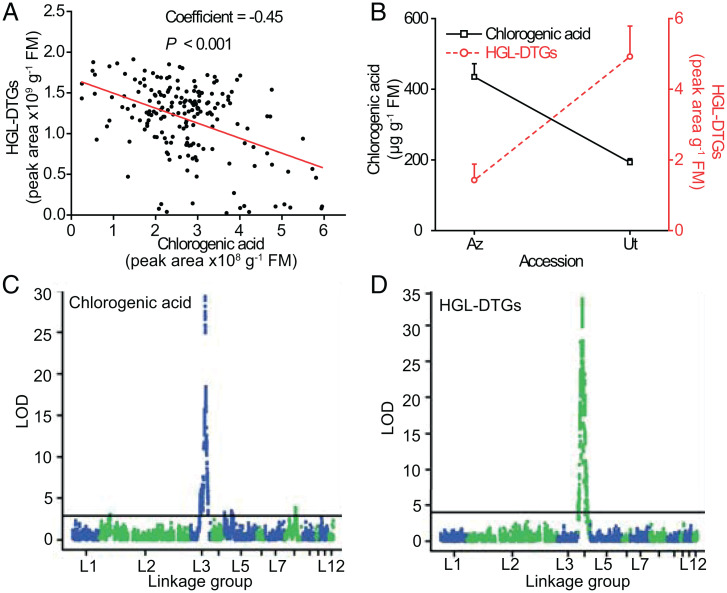
The abundances of CA and HGL-DTGs are genetically negatively correlated in natural accessions of *N. attenuata* and impute different loci in a RIL population. (*A*) Relative abundances of CA and HGL-DTGs in leaves of 183 natural accessions of *N. attenuata* are negatively correlated. (*B*) Abundances (mean + SE, *n* = 5) of CA and HGL-DTGs in two well-studied accessions of *N. attenuata*, Ut and Az. The QTL mapping using an AI-RIL population generated from intercrosses of the Ut and Az accessions and subsequent inbreeding revealed separate loci regulating the abundances of CA (*C*) and HGL-DTGs (*D*). Three genes imputed in the CA QTL—namely, a *MYBb308* transcription factor (TF), a *PAL* biosynthetic gene, and a bHLH TF—underwent VIGS, but only the MYB308 TF significantly altered CA contents (*SI Appendix*, Fig. S6).

*N. attenuata* plants have likely interacted with *M. sexta* larvae for the majority of their 24 million years of existence (32). To evaluate if natural populations harbor the expected signatures of a strong negative defensive synergy driven by a specialist herbivore’s detoxification strategy, we quantified CA and HGL-DTGs in 183 natural accessions of *N. attenuata* derived from seeds collected in the Great Basin Desert (33). Interestingly, there was a significant negative relationship between the abundances of CA and HGL-DTGs in leaves (Fig. 4*A*). Among these accessions, two well-studied accessions originate from Utah (Ut) and Arizona (Az), respectively. The absolute concentration of CA and HGL-DTGs in Ut and Az plants were quantified: Ut plants accumulated higher levels of HGL-DTGs and lower levels of CA, while Az plants showed the opposite pattern (Fig. 4*B*). At higher altitudes, with higher ultraviolet B (UV-B) fluence, plants from the Az accession would be selected for higher CA levels due to well-established sunscreen functions of CA (34). However, due to the detoxification process described here, Az plants would also be selected for lower HGL-DTG levels (Fig. 4*B*). Considering the more robust defense function of HGL-DTGs than CA (Fig. 3*C*), Az plants would be expected to be more susceptible to herbivore attack than UT plants. Az plants are also known to have higher nicotine levels and a disabled proteinase inhibitor defense system (35, 36), which may help to compensate for their lower HGL-DTG levels.

To elucidate the possible genetic basis underlying this negative correlation, we performed a quantitative trait locus (QTL) analysis using an advanced intercross recombinant inbred line (AI-RIL) population developed by crossing Ut and Az plants (37). The QTL mapping analysis revealed that the loci controlling CA and HGL-DTGs are on linkage groups 3 and 4, respectively (Fig. 4 *C* and *D*), of the published assembly of the *N. attenuata* genome (38). Genetic analysis of the genomic loci imputed by CA levels uncovered three candidate genes: a MYB (myeloblastosis virus) transcription factor, a basic helix-loop-helix (bHLH) transcription factor, and an upstream biosynthetic gene of CA, phenylalanine ammonia-lyase (PAL). Sequence analysis revealed that the MYB transcription factor, namely, *NaMYB308*, contained a premature stop codon in Ut plants but that Az plants had an intact coding region (*SI Appendix*, Fig. S6*B*). VIGS was performed in Ut and Az plants to specifically silence the candidate genes, and only silencing *NaMYB308* expression abolished the differences in CA contents between Ut and Az plants (*SI Appendix*, Fig. S6 *C* and *D*). Thus, *NaMYB308* likely controls CA accumulation differences between Ut and Az plants. Although a single sharp LOD (logarithm of odds) peak was imputed by the HGL-DTG contents, we were unsuccessful in identifying a protein-coding locus responsible for the differences in HGL-DTGs between Ut and Az plants. Previous research revealed that HGL-DTGs are positively regulated by jasmonate (JA) signaling (22), and here, we show that JA negatively regulates CA in Az plants through its influence on *NaMYB308* expression (*SI Appendix*, Fig. S6), suggesting that the covariance of CA and HGL-DTGs is regulated by JA signaling. While substantially more research is required to flesh out the mechanisms and evolutionary history of this negative genetic covariance, these results are consistent with its association with the detoxification strategy of *Manduca* larvae described here.

### Conclusion.

**Fig. 5.**
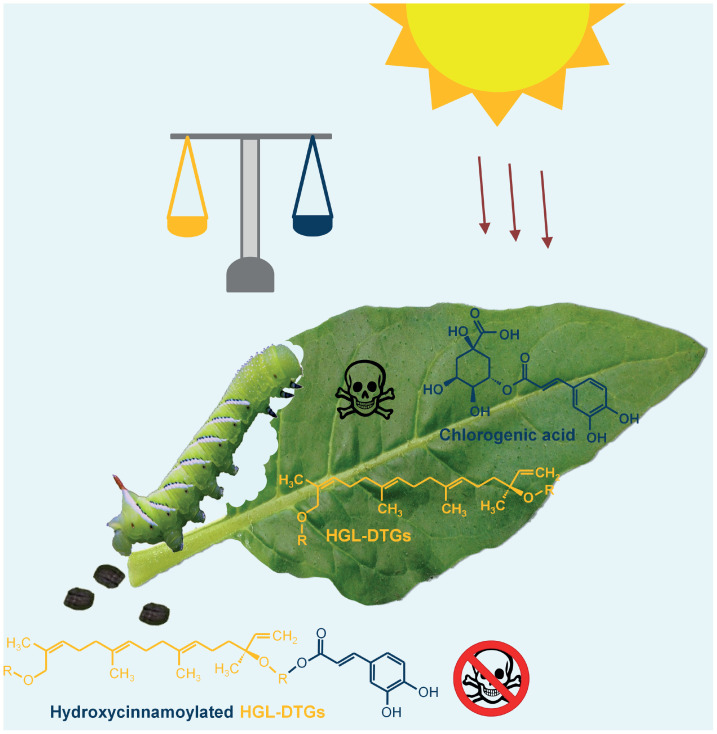
Postingestive interactions between diterpenoids and phenolics allow caterpillars to detoxify both defense pathways, while plants balance opposing selection pressures to minimize this detoxification process. Plants produce diverse specialized metabolites to deal with biotic and abiotic stresses from environments, such as diterpenoids (yellow) and phenolic CA (blue), both of which have deterrent effects on caterpillar feeding. Phenolics also function as sunscreens that protect plants from UV-B radiation. When fed these two toxic compounds, larvae of specialized insects can reesterify the phenolic moiety of CA onto the diterpenoid defenses and prevent the antiherbivore function of both defense compound classes. In nature, plants can balance the abundances of diterpenoids and phenolics to circumvent the detoxification of specialized insects and optimize UV protection.

The benefits of diversity in plant-specialized metabolites have long been recognized in early theoretical deliberations (6, 7, 39), and the synergistic effects of deploying mixtures are thought to accelerate the evolution of diversification (40). Through the study of frass metabolites, we found that the caffeoyl moieties of CA are reesterified to glucose of HGL-DTGs and reduce the growth-retardant effects of the two distinct metabolisms. This study presents a downside to metabolic diversity, which through the relentless evolutionary arms races among plants and their attackers could counter selection for enhanced metabolic diversity (Fig. 5).

## Method Details

### Plants and Growth Conditions.

The 31st inbred generation of *N. attenuata* was used as Ut WT genotype plants. Seeds were germinated on a mixture of plant agar with Gamborg’s B5 medium in sterile Petri dishes, and seedlings were transferred to pots and grown under 19 to 35 °C with 16 h of light (supplemental lighting by Philips Sun-T Agro 400- and 600-W sodium lights) and 60 to 65% relative humidity as previously described (41). Stably transformed and fully characterized RNA interference plants, including irUGT91T1 [A-11-538-05 (23)], irUGT74P5 [A-11-544-11 (23)], irUGT74_3&5 [A-12-076-07 (23)], irGGPPS [A-08-230-5 (22)], irHQT [A-14-153-6 (15)], and irMYB8 [A-07-810 (14)], were germinated and grown under glasshouse conditions. Cross plants were produced by crossing the homozygous irGGPPS and irHQT in both parental directions. Hemizygous irGGPPS and irHQT were produced by crossing the homozygous irGGPPS and irHQT with EV (A-03-009-1). The VIGS procedure was used with Ut WT plants and Az plants of the 22nd inbred generation (42), following the procedures described by Galis et al. (43). Briefly, leaves of young rosette-stage plants were pressure infiltrated with a mixture of *Agrobacterium tumefaciens* containing pBINTRA and either pTV-MYB308 or pTV00 (EV control). The VIGS experiments for NaCYP736A on EV or irGGPPS plants are described in Li et al. (18). VIGS experiments were repeated at least three times.

### *M. sexta* Growth Conditions and Larval Performance Assays.

*M. sexta* eggs were obtained from an in-house colony in which insects are reared in a growth chamber (Snijders Scientific, Tilburg, the Netherlands) at 26 °C:16-h light and 24 °C:8-h dark, 65% relative humidity, until hatching.

To measure the performance of *M. sexta* larvae on plants (EV, irHQT, irGGPPS, and Cross), a single newly hatched neonate was introduced onto the first stem leaf of each early elongated *N. attenuata* plant and allowed to feed freely. The larvae were weighed 4 d after release.

AD assays were performed in plastic polyethylen (PE)-packing cups containing AD based on the recipes of Machado et al. (44). Additionally, neonates were fed AD containing 2 mg g^−1^ fresh weight (FW)**-**purified HGL-DTGs (aqueous in 5 mL), 3.4 µmol g^−1^ FW CA (aqueous in 5 mL), and a combination of 2 mg g^−1^ FW HGL-DTGs and 3.4 µmol g^−1^ FW CA. The AD was exchanged with fresh diet after each weighing.

### Frass Collection.

Two freshly hatched neonates of *M. sexta* were reared on the first fully elongated stem leaf (S_1_; younger leaves are subsequently labeled S_2_, S_3_, and S_4_ proceeding up the main stalk) of transgenic 43-d-old flowering *N. attenuata* plants and retained on the S_1_ leaf with clip cages for 3 d. Approximately, 10 to 20 mg of frass were collected either from the top of the leaves with forceps or from the bottom of the clip cage. Frass from AD was collected using forceps from the PE-packing cups. All materials were flash frozen in liquid nitrogen and stored at −80 °C until use. In general, frass was collected from caterpillars while they were in the first to third instars.

### Extraction and Analysis of CA, HGL-DTGs, and HC-HGL-DTGs.

Plant samples were ground in liquid nitrogen to a fine powder and ca. 100 mg of leaf tissue were aliquoted for extraction. CA and HGL-DTGs were extracted using 1 mL 80% methanol aqueous buffer in a 2-mL Eppendorf tube containing two steel balls and were agitated twice at 1,200 strokes min^-1^ for 60 s using a GenoGrinder 2000. Homogenized samples were centrifuged twice at 16,000 × *g* for 20 min at 4 °C, and the supernatants were analyzed on a micrOTOF-Q II or Impact II system (Bruker) as previously described (19).

Collected frass was weighed and extracted using 80% aqueous methanol with the ratio of 1 mL per 100 mg or 1 mL per 10 mg of frass (experiment using the cross) and analyzed using the same method used for plant samples.

### Rapid Screening of HC-HGL-DTGs.

Chromatographic separation was performed using a Dionex UltiMate 3000 rapid separation LC system (Thermo Fisher), combined with a Thermo Acclaim RSLC 120 C_18_ column (particle size, 2.2 µm; average pore diameter, 120 Å; column dimension, 2.1 × 150 mm). Solvent A: water with 0.1% acetonitrile and 0.05% formic acid, and solvent B: acetonitrile with 0.05% formic acid, was used. Sample elution steps were as follows: 0 to 3 min at 10% solvent B, 3 to 12 min at 20% solvent B, 12 to 17 min at 35% solvent B, 17 to 23 min at 40% solvent B, 23 to 25 min at 45% solvent B, 25 to 30 min at 50% solvent B, 30 to 40 min at 90% solvent B, and 40 to 45 min at 90% solvent B, followed by column equilibration steps and a return to the starting conditions. A second gradient was also used with the same solvents for more rapid screening (experiment using the cross). Sample elution steps were as follows: 0 to 0.5 min at 10% solvent B, 0.5 to 23.5 min at 90% solvent B, and 23.5 to 25 min at 90% solvent B, followed by column equilibration steps and a return to the starting conditions. The injection volume was 2 µL, and the flow rate was 0.4 mL min^-1^.

MS detection was performed using a micrOTOF-Q II and an Impact II ultra high resolution quadrupole time-of-flight mass spectrometer (UHR-Q-TOF-MS) system, all equipped with an electrospray ionization (ESI) source and operated in positive ion mode. ESI conditions for the micrOTOF-Q II system were endplate offset of 500 V, capillary voltage of 4,500 V, capillary exit of 130 V, dry temperature of 180 °C, and dry gas flow of 10 L min^−1^. ESI conditions for the Impact II UHR-Q-TOF-MS system were endplate offset of 500 V, capillary voltage of 4,500 V, nebulizer of 2 bar, dry temperature of 200 °C, and dry gas flow of 8 L min^−1^. MS data were collected over a range of *m/z* 100 to 1,600. Mass calibration was performed using sodium formate (50 mL of isopropanol, 200 µL of formic acid, and 1 mL of 1 M NaOH in water). Data files were calibrated using the Bruker high-precision calibration algorithm.

MS/MS experiments were performed using AutoMS/MS runs at various collision-induced dissociation (CID) voltages from 12 to 25 eV for HGL-DTGs and HC-HGL-DTGs. Instrument control, data acquisition, and reprocessing were performed using HyStar 3.1 (Bruker Daltonics). Peak areas were integrated with QuantAnalysis (Bruker Daltonics).

### Dereplication and Annotation of HGL-DTGs.

The dereplication workflow relies on a well-established and extensive MS and MS/MS database constructed from previously identified HGL-DTGs from 35 solanaceous species (19). It includes a detailed rule set for the annotation of fragmentation patterns of the different moieties decorating the 17-hydroxygeranyllinalool (17-HGL) aglycone. To visualize HGL-DTG profiles, we computed the extracted ion chromatogram (EIC) of *m/z* 271.2420, which corresponds to the 17-HGL aglycone fragment lacking both hydroxyl groups at the C-3 and C-17 positions. The definition of malonylated HGL-DTGs or core HGL-DTGs is based on whether the compounds contain malonyl moieties (20). The identification levels are based on community standards reported in Sumner et al. (45).

### Isolation of HGL-DTGs from *N. attenuata* Plants.

*N. attenuata* leaf material (500 g) was ground in liquid nitrogen and extracted in 2.5 L of 80% methanol overnight. Further extraction steps were performed as previously described (18). The supernatants were fractionated by reversed-phase fast protein liquid chromatography (Pharmacia Biotech LCC 501 Plus Core) at a flow rate of 5 mL min^-1^. Mixtures of solvent A (Millipore water) and solvent B (methanol, gradient grade; Merck) were used to elute analytes from the column as follows: 0 to 10 min at 20% of solvent B, 10 to 15 min at 40% of solvent B, 15 to 25 min at 40% of solvent B, 25 to 30 min at 60% of solvent B, 30 to 40 min at 60% of solvent B, 40 to 45 min at 80% of solvent B, 45 to 55 min at 80% of solvent B, 55 to 60 min at 100% of solvent B, 60 to 85 min at 100% of solvent B, and 85 to 95 min at 100 to 0% of solvent B. Highly enriched fractions of HGL-DTGs were collected from fractions 15 to 17 at ∼50 to 60% methanol; these were subsequently dried to produce about 220 mg of HGL-DTG crude mixture. The mixture was used for *M. sexta* bioassays on irGGPPS plants and AD.

### Purification of Caffeoylated HGL-DTG from *M. sexta* Frass.

Frass of *M. sexta* larvae reared on *N. attenuata* WT plants was collected and purified using high performance liquid chromatography (HPLC) as previously described for the hydroxylated HGL-DTGs (18). The HPLC fractions containing caffeoylated HGL-DTGs were further separated using the same HPLC systems with HPLC-grade acetonitrile (VWR International) as solvent B instead of methanol and the following chromatographic solvent gradient: 0 to 3 min at 42% of solvent B, 3 to 11 min at 50% of solvent B, 11 to 15 min at 98% of solvent B, 15 to 17 min at 98% of solvent B, and 17 to 20 min at 42% of solvent B. The obtained pure metabolites were used for NMR analysis.

### NMR Analysis.

^1^H NMR, ^13^C NMR, ^1^H-^1^H correlation spectroscopy (COSY), ^1^H-^13^C heteronuclear single quantum correlation (HSQC), ^1^H-^13^C heteronuclear single quantum correlation - total correlation spectroscopy (HSQC-TOCSY), and ^1^H-^13^C heteronuclear multiple bond correlation (HMBC) experiments were measured on Bruker 500-MHz Avance III HD and 700-MHz Avance III HD NMR spectrometers (Bruker Biospin, Germany). A 5-mm proton-optimized triple resonance NMR ‘inverse’ (TCI) cryoprobe (500 MHz) or a 1.7-mm TCI cryoprobe (700 MHz) at a probe temperature of 298 K was used. Samples were prepared in methanol-d_3_, and ^1^H and ^13^C chemical shifts (δ) were referenced to the residual solvent signals of methanol-d_3_ at δ_H_ 3.31 and δ_C_ 49.15, respectively.

### Correlation of CA and HGL-DTGs in *N. attenuata* Natural Population.

For the analysis of HGL-DTGs and CA in 183 accessions, we mined the published data by Li et al. (33). The correlation between HGL-DTGs and CA was performed using Pearson correlation across all 183 accessions. Significance levels for correlation values (*r*) were determined following the number of metabolite pairs (*n*) using the equation *t* = *r* × (*n* − 2)^0.5^/(1 − *r^2^*)^0.5^, with *t* indicating t-value required for the test of significance.

### QTL Mapping.

We used a biparental (Ut and Az parental genotypes) AI-RIL population previously described by Zhou et al. (37) to map the genetic loci underlying the variation in CA and HGL-DTG levels. CA and HGL-DTG tissue concentrations were analyzed in fully developed stem leaves of elongated nonflowering plants in the F_11_ generation (five generations of inbreeding after the F_2_ to F_6_ intercrossing procedure) (37). Three 6-mm leaf discs (average fresh mass of 26 ± 3 mg per leaf disc) were harvested from each of the 261 recombinant inbred line (RIL) plants, flash frozen in liquid nitrogen, extracted, and analyzed on micrOTOF-Q II as described above.

QTL mapping was performed on the fresh mass-normalized peak area data for total HGL-DTGs and CA after log transformation using the QTLRel package (46) in R version 3.3.1 as described by Zhou et al. (37).

### Quantitative Real-Time PCR Analysis of Transcript Levels.

Total RNA was extracted from leaves of VIGS *N. attenuata* plants using the Plant RNA purification kit (Macherey-Nagel) according to the manufacturer’s instructions. Complementary DNA (cDNA) libraries were prepared using the PrimeScript RT Reagent kit (Takara Bio Inc., Japan). Quantitative real-time PCR (qRT-PCR) were performed on a Stratagene 500 MX3005P (Agilent) using a SYBR green reaction mix (Euro-gentec, Belgium) in accordance with the manufacturer’s instructions. The mRNA of the *N. attenuata* eukaryotic translation initiation factor 5α-2 gene (*IF5α-2*,NIATv7_g37283) was used as internal control. The delta-delta Ct method was used for data analysis (47). The amplification specificity of primers was confirmed by single peaks in a dissociation curve following qRT-PCR. Primers used in this project are listed in *SI Appendix*, Table S1.

### Statistical Analysis.

Data were analyzed using SPSS 20.0 (SPSS Inc.). Unless otherwise stated, parametric data were compared using ANOVA followed by Honestly Significant Difference (HSD) or Least Significant Difference (LSD) tests as described in figure legends of corresponding data.

## Supplementary Material

Supplementary File

## Data Availability

MS metabolomics data were submitted and have been deposited in the MetaboLights public database (accession no. MTBLS3676) (48). All other study data are included in the article and/or *SI Appendix*.
