# Supplementary material for: The downside of metabolic diversity: Postingestive rearrangements by specialized insects
Source: Proc Natl Acad Sci U S A. 2022 Jun 6;119(24):e2122808119. doi: 10.1073/pnas.2122808119 (PMC9214519; doi:10.1073/pnas.2122808119)
Supplement: Supplementary File [file pnas.2122808119.sapp.pdf]

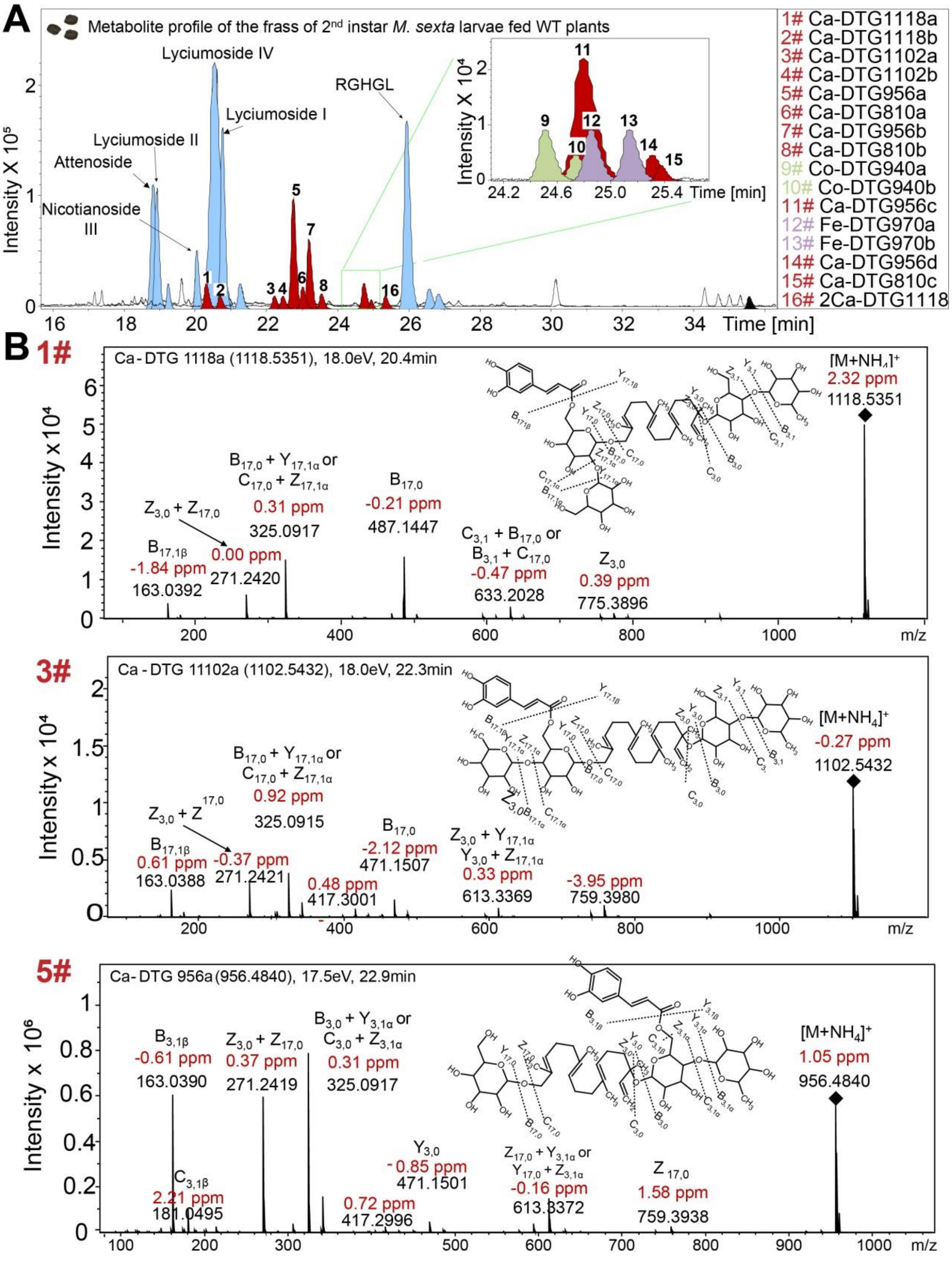
A
Metabolite profile of the frass of 2nd instar M. sexta larvae fed WT plants
Lyciumoside IV
Lyciumoside I
Lyciumoside II
Attenoside
Nicotianoside III
RGHGL
Intensity X 10^5
Time [min]
Intensity X 10^4
24.2 24.6 25.0 25.4 Time [min]
1# Ca-DTG1118a
2# Ca-DTG1118b
3# Ca-DTG1102a
4# Ca-DTG1102b
5# Ca-DTG956a
6# Ca-DTG810a
7# Ca-DTG956b
8# Ca-DTG810b
9# Co-DTG940a
10# Co-DTG940b
11# Ca-DTG956c
12# Fe-DTG970a
13# Fe-DTG970b
14# Ca-DTG956d
15# Ca-DTG810c
16# 2Ca-DTG1118
B
1#
Ca-DTG 1118a (1118.5351), 18.0eV, 20.4min
Intensity x10^4
[M+NH4]+ 2.32 ppm 1118.5351
B17,1β -1.84 ppm 163.0392
Z3,0 + Z17,0 0.00 ppm 271.2420
B17,0 + Y17,1α or C17,0 + Z17,1α 0.31 ppm 325.0917
B17,0 -0.21 ppm 487.1447
C3,1 + B17,0 or B3,1 + C17,0 -0.47 ppm 633.2028
Z3,0 0.39 ppm 775.3896
m/z
3#
Ca-DTG 11102a (1102.5432), 18.0eV, 22.3min
Intensity x 10^4
[M+NH4]+ -0.27 ppm 1102.5432
B17,1β 0.61 ppm 163.0388
Z3,0 + Z17,0 -0.37 ppm 271.2421
B17,0 + Y17,1α or C17,0 + Z17,1α 0.92 ppm 325.0915
0.48 ppm 417.3001
B17,0 -2.12 ppm 471.1507
Z3,0 + Y17,1α Y3,0 + Z17,1α 0.33 ppm 613.3369
-3.95 ppm 759.3980
m/z
5#
Ca-DTG 956a (956.4840), 17.5eV, 22.9min
Intensity x 10^6
[M+NH4]+ 1.05 ppm 956.4840
B3,1β -0.61 ppm 163.0390
Z3,0 + Z17,0 0.37 ppm 271.2419
B3,0 + Y3,1α or C3,0 + Z3,1α 0.31 ppm 325.0917
C3,1β 2.21 ppm 181.0495
0.72 ppm 417.2996
Y3,0 -0.85 ppm 471.1501
Z17,0 + Y3,1α or Y17,0 + Z3,1α -0.16 ppm 613.3372
Z17,0 1.58 ppm 759.3938
m/z

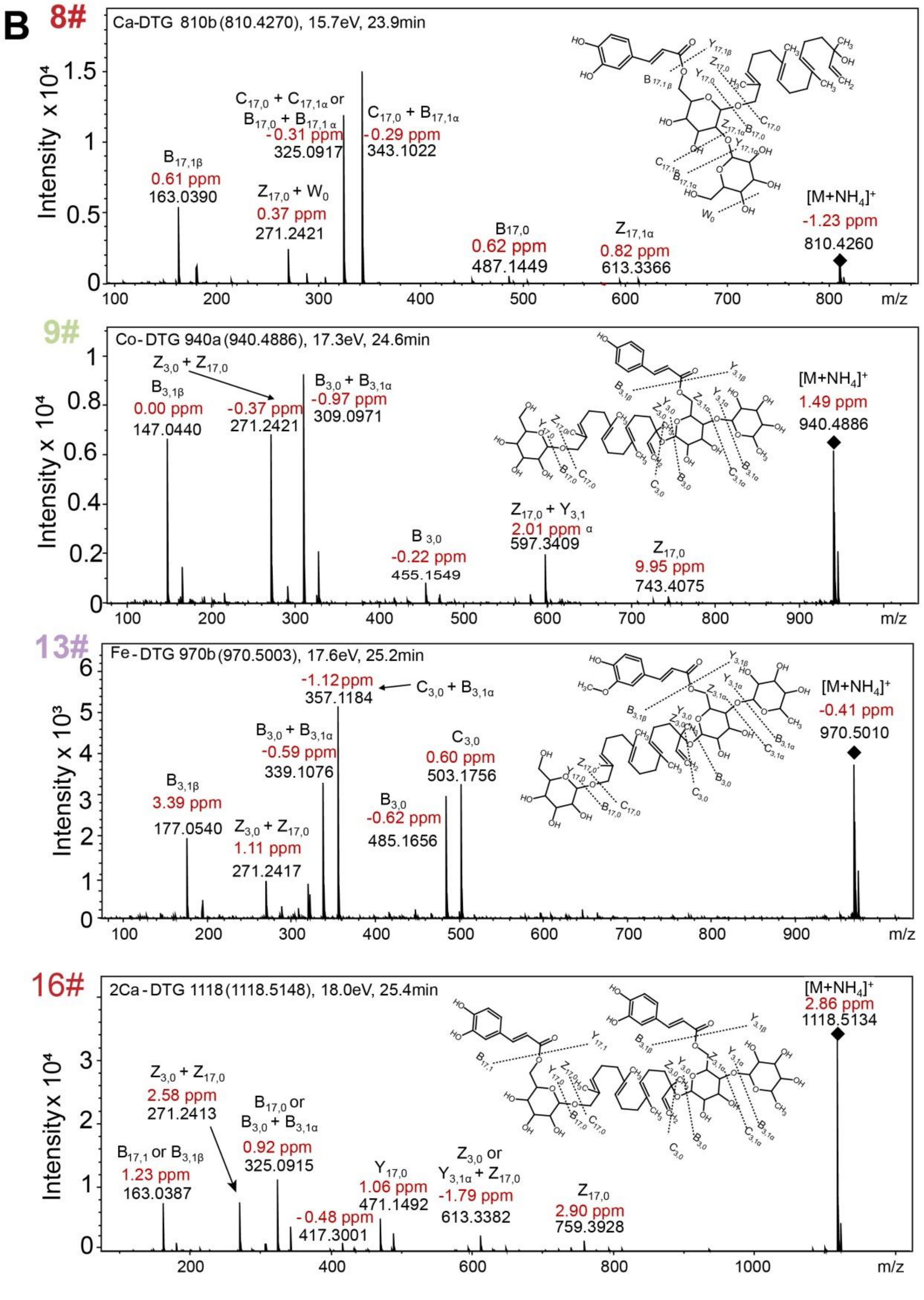

Figure S1: Characterization and annotation of novel HC-HGL-DTGs via MS/MS in the frass of *M. sexta* larvae fed WT *N. attenuata* plants

(**A**) EIC traces of *m/z* 271.2420 for the 17-HGL aglycone representing the metabolic decoration of the HGL-DTG profile in the frass of *M. sexta* larvae fed WT *N. attenuata* plants. Novel hydroxycinnamoylated HGL-DTG (HC-HGL-DTG) products are highlighted in red, green and purple for caffeoylated HGL-DTGs (Ca-DTGs), coumaroylated HGL-DTGs (Co-DTGs) and feruloylated HGL-DTGs (Fe-DTGs), respectively. The 16 detected HC-HGL-DTGs are numbered

following their retention times. The number in their name, such as the "1118" in compound 1#, indicates the *m/z* of the ammonium adducts. Different isomers are distinguished by a suffix, such as the "a" in compound 1#. (**B**) The associated MS/MS spectra for selected de-glycosylation products and HC-HGL-DTGs are displayed. A putative structure is assigned to all selected compounds and the fragmentation scheme is explained via the predicted neutral losses from the ammonium adduct of the molecular ion $[M+NH_4]^+$.

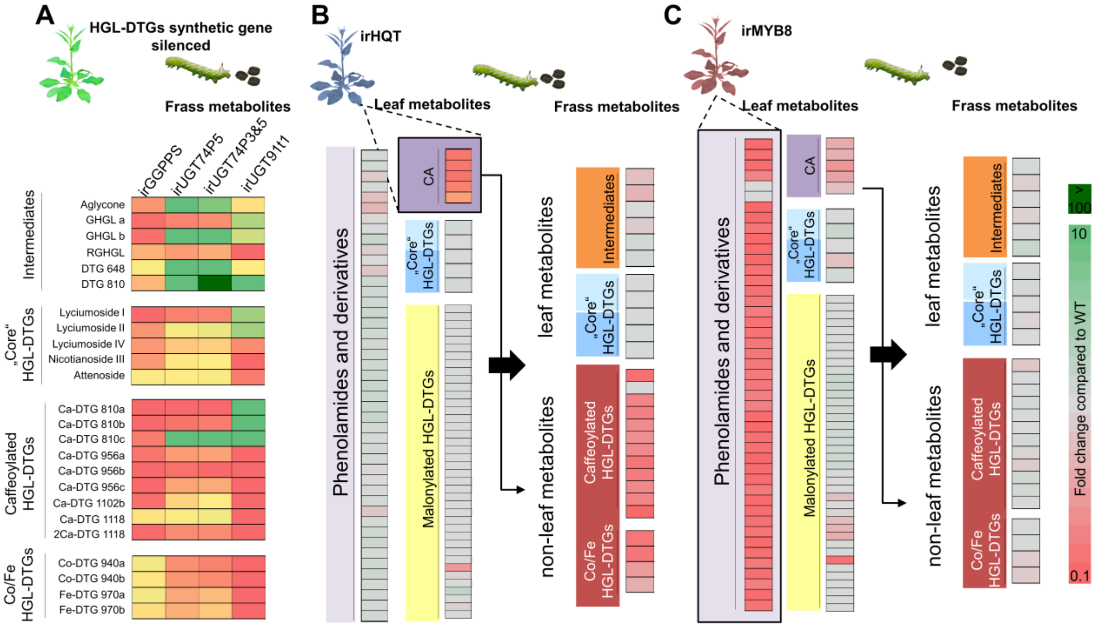

Figure S2. The HC-HGL-DTGs discovered in the frass of *M. sexta* larvae originate from ingested HGL-DTGs and CA in leaves

(**A**) Heat map of HGL-DTG-related metabolites in the frass of *M. sexta* larvae that fed on different previously published HGL-DTG biosynthetic gene-silenced plants: irGGPPS plants are silenced in total HGL-DTG accumulations; irUGT74P5, irUGT74P3&5, and irUGT91T1 are silenced in different combinations of the UGTs that add glucose residues to the diterpene backbone during HGL-DTG biosynthesis. Metabolites in *N. attenuata* leaves (left) and frass (right) of *M. sexta* that fed on irHQT plants, which are silenced in CA biosynthesis (**B**) and irMYB8 plants, which are silenced in a TF that regulates phenolamide biosynthesis (**C**).

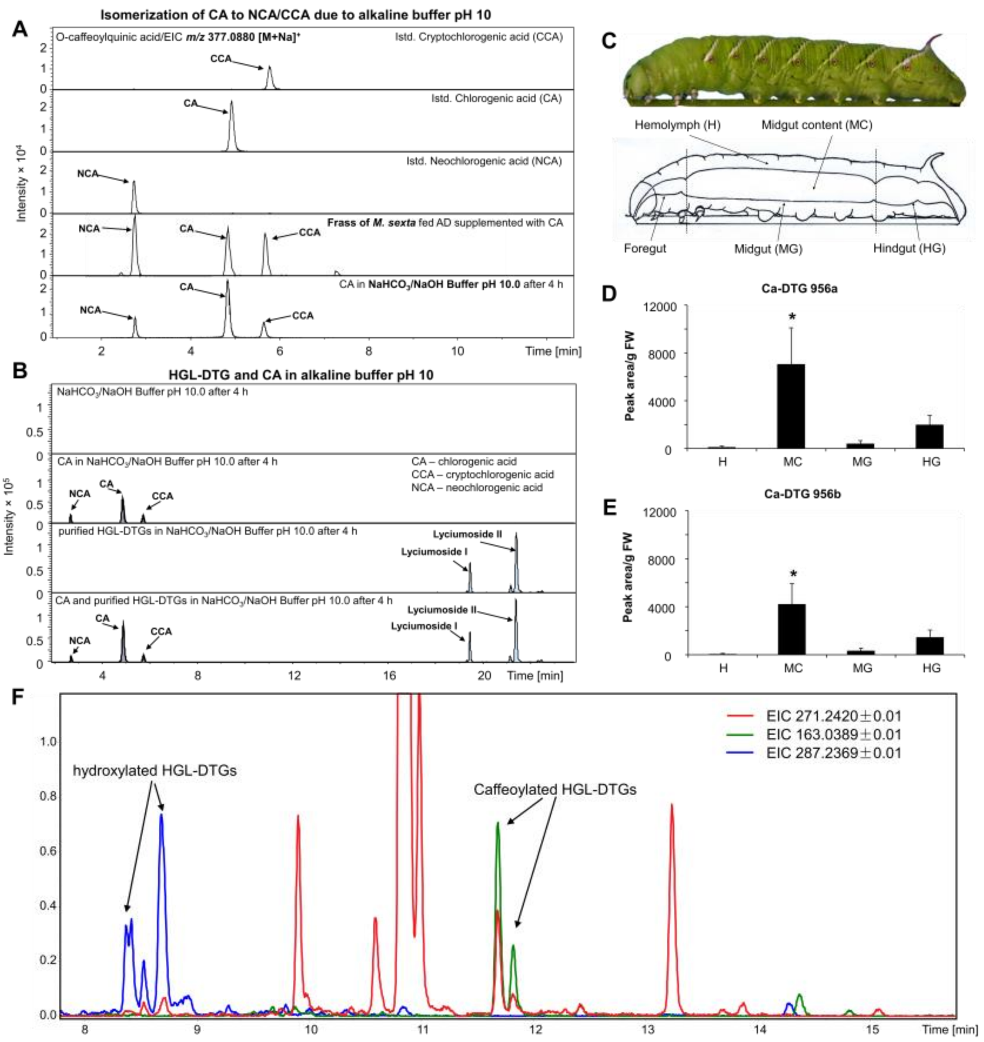

Figure S3: Isomerization and localization of caffeoylated HGL-DTGs in *M. sexta* larvae
(**A**) CA will isomerize into cryptochlorogenic acid (CCA) and neochlorogenic acid (NCA) in alkaline sodium carbonate buffers (pH = 10) and in the guts of *M. sexta* larvae. (**B**) Other than the isomerization of CA s, no additional non-catalytic reactions were detected when CA and HGL-DTGs were combined in alkaline buffers. (**C**) Schema of the different parts sampled from *M. sexta* larvae that included hemolymph (H), midgut (MG) and hindgut (HG) tissues, as well as the midgut content (MC). The relative abundances (mean + SE, *n* = 4) of caffeoylated HGL-DTG 5# (Ca-DTG 956a, **D**) and caffeoylated HGL-DTG 7# (Ca-DTG 956b, **E**) in different parts of 4th instar *M. sexta* larvae that fed on *N. attenuata* plants. The annotations of the two compounds are shown in Figure S1. (**F**) EIC traces of *m/z* 271.2420 (aglycone fragment of HGL-DTGs), *m/z* 163.0389 (caffeoyl fragment) and *m/z* 287.2369 (aglycone fragment of hydroxylated HGL-DTGs) representing the metabolic profile in the frass of *M. sexta* larvae fed WT *N. attenuata* plants. The lack of co-eluting peak signals of EIC *m/z* 163.0389 and *m/z* 287.2369 reveals the absence of hydroxylated HC-HGL-DTGs. Asterisks indicate significantly different tissues concentrations (*, *P*<0.05, one-way ANOVA followed by Tukey's HSD *post-hoc* tests).

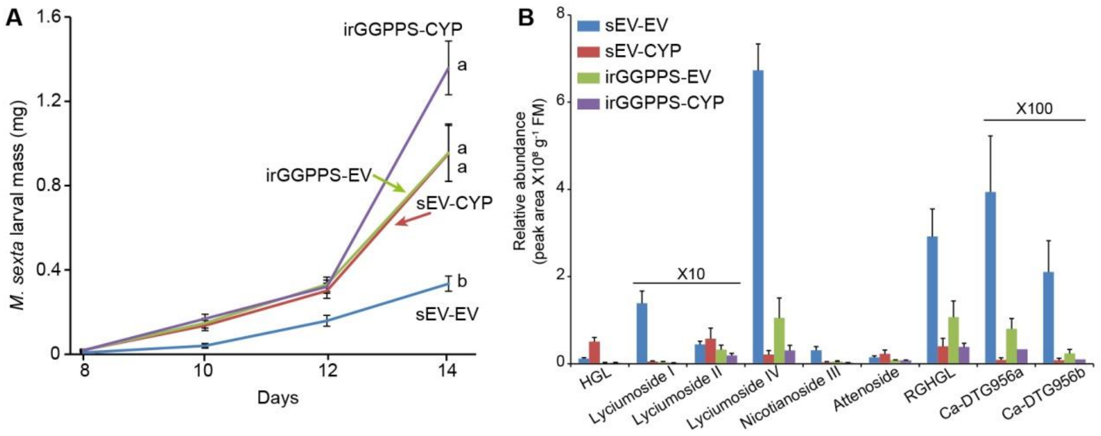

Figure S4. Silencing HGL-DTG biosynthetic genes affect *M. sexta* performance and HGL-DTG frass profiles of caterpillars fed these silenced plants

(**A**) VIGS vectors with a fragment of the HGL-DTGs biosynthetic gene *NaCYP736A* (CYP) and the empty vector (EV) were independently infiltrated into the stably transformed plants, irGGPPS and empty vector (stably transformed EV, sEV). Mass (mean ± SE, n = 10-14) of *M. sexta* larvae fed different plants were determined at the indicated times. (**B**) The abundance (mean + SE, n = 6) of HGL-DTG-related metabolites in the frass of *M. sexta* larvae that fed on indicated plants. “X10” and “X100” above some metabolites indicate 10- and 100-fold magnifications of the intensities to enhance visibility. Different letters indicate significantly different tissues concentrations (*, P<0.05, one-way ANOVA followed by Tukey’s HSD *post-hoc* tests).

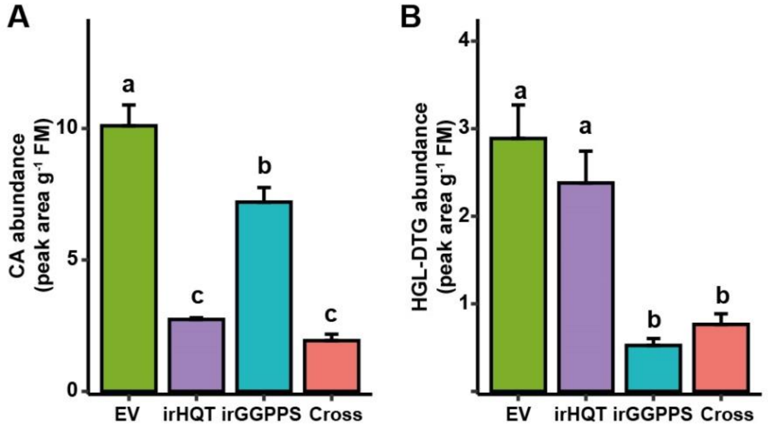

Figure S5. The abundance of CA and HGL-DTGs in the leaves of different transgenic plants

The relative abundances (mean + SE, n = 5) of CA (**A**) and HGL-DTGs (**B**) in the leaves of hemizygous irHQT, the hemizygous irGGPPS and the hemizygous Cross plants, with the homozygous empty vector- transformed (EV) as control. Different letters indicate significant differences ($P < 0.05$, one-way ANOVA followed by Tukey's *post-hoc* test).

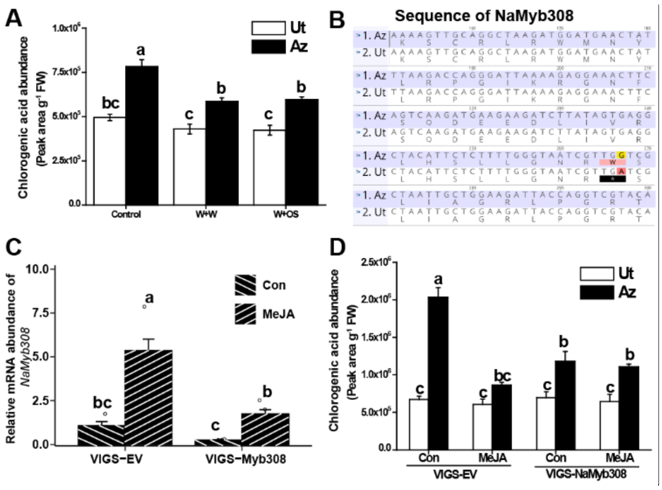

Figure S6. NaMyb308 contributes to different accumulations of CA between Ut and Az accessions

(**A**) Relative abundance (mean ± SE, n = 5) of chlorogenic acid (CA) in the leaves of Ut and Az *N. attenuata* accession plants that were elicited by treating freshly produced puncture wounds in leaves with water (W+W) or oral secretion of *M. sexta* larvae (W+OS). (**B**) Sequence comparisons of the NaMyb308 in Ut and Az. A single nucleotide difference at position +267 leads to a premature stop codon in the Ut accession. (**C**) Relative mRNA abundance (mean + SE, n = 4-5) of *NaMyb308* in the control (Con) or MeJA-treated (MeJA) leaves on Az VIGS plants. VIGS of NaMyb308 (VIGS-Myb308) versus a control VIGS empty vector (VIGS-EV) were infiltrated in Az plants. (**D**) Relative abundance (mean + SE, n = 4-5) of CA in control or MeJA-treated Ut and Az VIGS plants. Different letters indicate significant differences among different transgenic plants ($p < 0.05$, one-way ANOVA followed by Tukey's HSD *post-hoc* tests).

Table S1. DNA primers used in this study.

Name	Sequence (5'-3')	Purpose
NaMyb308-v-F	GCGGCGGTCGACTTACATTGAGTACTGCTACTTG	VIGS construct
NaMyb308-v-R	GCGGCGGGATCCGATTTTGCTCCTTTTGAT	VIGS construct
NaMyb308-q-F	TGTGCTGGAATTGAACCAAACC	RT-qPCR
NaMyb308-q-R	GATGGGCTTCGGGGCAAATA	RT-qPCR
NaIF5a-q-F	GTCGGACGAAGAACACCATT	RT-qPCR
NaIF5a-q-R	CACATCACAGTTGTGGGAGG	RT-qPCR
